# The Nordrach Sanatorium

**Published:** 1902-10-11

**Authors:** 


### THE NORDRACH SANATORIUM.

Perhaps no Continental sanatorium for consumption is so well and so favourably known in England as that at Nordrach, conducted by Dr. Hettinger. The sanatorium is a fine building in the Renaissance style of architecture. It is surrounded by pine woods, and, being in a valley, is sheltered from the most trying winds in winter, while in summer the temperature is moderate. The climatic conditions of the sanatorium and its immediate neighbourhood are thus so equable that invalids can remain in the open air without discomfort until the evening, and even during the winter months the temperature rarely falls many degrees below freezing point. One important condition is that the air is absolutely free from dust.

Like all other sanatoria for the treatment of consumption, Nordrach has the rooms occupied by patients facing south. They are provided with large windows, and, in accordance with correct hygienic principles, the furniture is simple. The total accommodation is for 40 patients.

The sanitary arrangements are of the best and most modern kind. The method of drainage adopted is that known as the "Fosse Mourass" system in which clarifying tanks are used. There is a central system of warming the rooms by hot-water coils.

The treatment of the patients is emphatically an individualised one, and in carrying it out, Dr. Hettinger is assisted by two other medical officers. So far as purely medical treatment goes it is limited to those cases in which some complication exists, or in which some distressing symptom calls for alleviation. Of course the diet is liberal and consists of five regular meals daily.

Although some of our English sanatoria are as fine as any in the world and are as well conducted in every way, it would seem to be certain that there is a percentage of cases who do better when placed amid entirely fresh surroundings, where the patient's thoughts are drawn more easily away from brooding on his condition, where the scenery is different, a foreign language spoken, and above all where he is far removed from fussy friends. For such cases there could hardly be a better place than Nordrach, and the patient should be prepared to devote at least three or four months to the cure.

The rates charged are from 8s. to 12s. a day, according to the size and position of the room chosen; but the diet and treatment are alike in all cases.

